# Rapid Amplification and Detection of Single‐Stranded Nucleic Acids for Point‐of‐Care Diagnosis

**DOI:** 10.1002/smtd.202401733

**Published:** 2025-01-06

**Authors:** Jinglin Fu, Qiaochu Zhang, Shiming Liu, Derek Puyat, Akshay Shah, Alireza Ebrahimimojarad, Sung Won Oh

**Affiliations:** ^1^ Department of Chemistry Rutgers University‐Camden Camden NJ 08102 USA; ^2^ Center for Computational and Integrative Biology Rutgers University‐Camden Camden NJ 08102 USA

**Keywords:** enzyme‐based nucleic acid detection, isothermal amplification, nucleic acid hybridization circuit, nucleic acid nanotechnology, point‐of‐care diagnostics, rapid nucleic acid detection

## Abstract

Nucleic acid detection plays a crucial role in various applications, including disease diagnostics, research development, food safety, and environmental health monitoring. A rapid, point‐of‐care (POC) nucleic acid test can greatly benefit healthcare system by providing timely diagnosis for effective treatment and patient management, as well as supporting diseases surveillance for emerging pandemic diseases. Recent advancements in nucleic acids technology have led to rapid assays for single‐stranded nucleic acids that can be integrated into simple and miniaturized platforms for ease of use. In this review, the study focuses on the developments in isothermal amplification, nucleic acid hybridization circuits, various enzyme‐based signal reporting mechanisms, and detection platforms that show promise for POC testing. The study also evaluates critical technical breakthroughs to identify the advantages and disadvantages of these methods in various applications.

## Graphical abstract

A rapid, point‐of‐care nucleic acid test can greatly benefit healthcare system by providing timely diagnosis for effective treatment and patient management, as well as supporting diseases surveillance for emerging pandemic diseases. Recent advancements in nucleic acids technology have led to rapid assays for single‐stranded nucleic acids that can be integrated into simple and miniaturized platforms for ease of use.

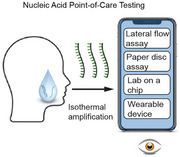

## 1 Introduction

**Figure 1:**
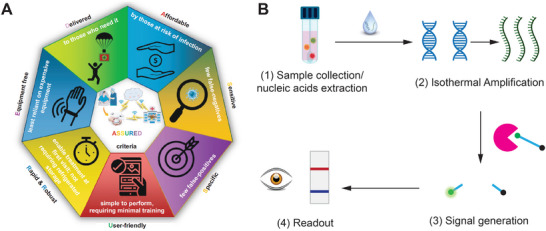
Guideline for POC testing of nucleic acids. A) World Health Organization's ASSURED Criteria for POC Diagnosis. Reproduced under the terms of the Creative Commons CC‐BY license.^[^ ^8^ ^]^ Copyright 2021, Springer Link. B) A general workflow for nucleic acid detection.

Clinical diagnosis is essential for identifying diseases, assessing medical conditions, formulating treatment plans, and evaluating their effectiveness. There is a significant demand for simplified diagnostic procedures and equipment to enhance accessibility and affordability for use. In the past few decades, the advancement of POC diagnosis has attracted people's attention, which aims to be performed at or near the patient's location, providing quick results.^[^ ^1^, ^2^ ^]^ POC tests do not require centralized laboratory processing and can be administered by healthcare professionals or by patients themselves. An even more accessible option is over‐the‐counter (OTC) tests, which can be sold directly to consumers without requiring a prescription.^[^ ^3^, ^4^, ^5^ ^]^ Examples of POC or OTC tests include rapid strep tests, pregnancy tests, and COVID‐19 antigen rapid tests. POC tests are particularly valuable in combating pandemic or epidemic diseases, as they offer rapid results and are easily accessible for tracking the spread of infectious diseases within the population. According to a recent report on Fortune Business Insights, the global market for POC diagnostics was valued at USD 30.87 billion in 2023. It is projected to grow from USD 31.57 billion in 2024 to USD 51.19 billion by 2032.^[^ ^6^ ^]^ To guide future development and implementation, the World Health Organization (WHO) has established “ASSURED” criteria for high‐quality POC tests (**Figure** **1**A), which should be “Affordable, Sensitive, Specific, User‐friendly, Rapid, Equipment‐free, and Deliverable”.^[^ ^7^, ^8^ ^]^ WHO highlights three essential characteristics of accuracy, accessibility, and affordability for a POC test. A test meeting these criteria will more likely support and improve patient outcomes by providing timely diagnoses, which is of particular interest to rural areas or undeveloped regions lacking necessary health infrastructure.

The COVID‐19 pandemic has created an urgent demand for a quick, simple, cost‐effective, and reliable assay for mass screening to help control and prevent the spread of disease infections. However, current diagnostic tools do not fully address this urgent need. The FDA (Food and Drug Administration)‐approved, OTC COVID‐19 antigen kit is marketed as a rapid test for home use, that detects antigen proteins from SARS‐COV‐2 virus. But this antigen test is less accurate and may generate more false negatives, particularly for individuals in the early stage of infection or those who are asymptomatic.^[^ ^9^, ^10^ ^]^ Serological antibody immunoassay can identify the presence of antibodies produced in response to infection, however, these assays do not directly indicate whether the infection is active or has been cleared from the body, leaving uncertainty about transmissibility.^[^ ^11^ ^]^ As a result, rapid antigen or antibody tests are primarily used for presumptive screening, necessitating follow‐up nucleic acid tests to confirm any positive results.^[^ ^12^ ^]^ Polymerase chain reaction (PCR) is considered the gold standard for diagnosing an active infection by directly detecting pathogenic DNA or RNA.^[^ ^13^ ^]^ PCR amplifies target DNA segments by two‐fold in each thermal cycle and can produce millions to billions of amplicons from a single DNA template after 20 to 40 cycles.^[^ ^14^ ^]^ Due to its high sensitivity, PCR can even detect a single copy of nucleic acids. While laboratory PCR tests can be completed in just a few hours, the actual turnaround time in clinical settings usually takes around 24 to 48 hours to deliver results to patients due to processes such as sample collection, transport, testing, and reporting summary. Consequently, conventional PCR does not satisfy the “ASSURED” criteria and fails to fulfill the growing demand for POC tests as required by public health, particularly in rural and underdeveloped areas.

To address the limitations of current molecular tests, significant efforts have been focused on developing new assays for rapid and robust nucleic acid detection.^[^ ^15^ ^]^ As illustrated in Figure 1B, a typical nucleic acid assay consists of four steps: 1) sample collection and nucleic acid extraction, 2) amplification of target strands, 3) signal generation, and 4) readout. The development of POC‐based nucleic acid tests has aimed at simplifying and integrating these steps. These advancements include rapid extraction,^[^ ^16^, ^17^ ^]^ isothermal amplification of nucleic acids,^[^ ^18^ ^]^ quick signal generation for sensitive detection, and innovative platforms designed for easy operation and signal readout. Although advancements in isothermal amplification can be applied to both single‐stranded DNA (ssDNA) and double‐stranded DNA (dsDNA), ssDNA or RNA molecules are more versatile for engineering mechanisms and circuits to amplify signals, making detection easier. Various nucleic acid‐based reaction circuits can efficiently detect ssDNA or RNA targets, producing highly sensitive and specific signals for detection, such as molecular beacons,^[^ ^19^ ^]^ toehold‐mediated strand displacement,^[^ ^20^ ^]^ DNA‐assembly circuitry,^[^ ^21^ ^]^ riboregulators,^[^ ^22^ ^]^ and CRISPR‐Cas technology.^[^ ^23^ ^]^

In this review, we highlight recent advancements in the rapid amplification and detection of single‐stranded nucleic acids that can be incorporated into POC platforms for disease diagnosis. We specifically examine developments in isothermal amplification, nucleic acid hybridization circuits, various enzyme‐based signal generation mechanisms, and detection platforms promising for POC testing. Additionally, we discuss the advantages and disadvantages of these methods in the clinical POC diagnosis of nucleic acids.

## 2 Isothermal Amplification of Single‐Stranded Nucleic Acids

Current clinical diagnostics require high sensitivity in nucleic acid detection, with a limit of detection (LoD) as low as 100 copies per ml (or attomolar concentrations).^[^ ^24^, ^25^, ^26^ ^]^ Some studies indicate that a 10‐fold increase in the LoD of the assay could result in a 13% rise in the false negative rate, potentially missing additional infected patients.^[^ ^26^ ^]^ To reach this level of sensitivity, it is often necessary to amplify target nucleic acids, producing amplicons that are millions to a billion of times greater than the original concentration. Isothermal amplification (IA) is a technique that allows for streamlined exponential amplification of target DNA or RNA at a constant temperature.^[^ ^18^ ^]^ Unlike conventional PCR, IA does not require thermal cycling for amplification, making the process simpler and faster.^[^ ^27^ ^]^ Thus, IA technology aligns with the “ASSURED” criteria for improving POC tests by offering improved sensitivity, affordability, and accessibility. Below, we briefly discuss some isothermal methods that show promise for rapid nucleic acid tests during the COVID‐19 pandemic.

**Figure 2:**
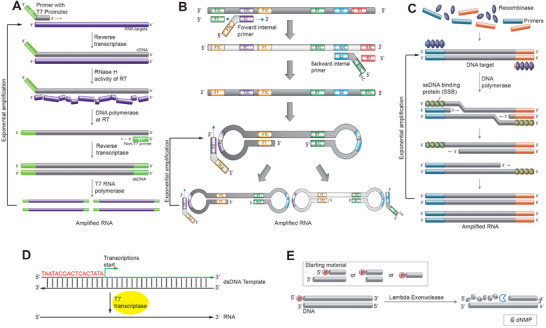
Examples of nucleic acid isothermal amplification. A) Principle of nucleic acid sequence‐based amplification (NASBA). Reproduced with permission.^[^ ^149^ ^]^ Copyright 2024, New England Biolabs. B) Principle of loop‐mediated isothermal amplification (LAMP). Reproduced with permission under a Creative Commons CC‐BY license.^[^ ^36^ ^]^ Copyright 2022, MDPI. C) Schematic illustration of recombinase polymerase amplification (RPA) technology. Reproduced with permission.^[^ ^150^ ^]^ Copyright 2024, New England Biolabs. D) T7 promoter‐initiated mRNA transcription from a dsDNA template. E) Degradation of dsDNA into ssDNA by lambda exonucleases using 5’ end phosphorylation. Reproduced with permission.^[^ ^151^ ^]^ Copyright 2024, New England Biolabs.

In **Figure** **2**A, “Nucleic Acids Sequence‐Based Amplification (NASBA)” is a primer‐dependent method that amplifies ssRNA targets using an enzyme cocktail that includes reverse transcriptase (RTase), Ribonuclease (RNase) H, and transcriptase.^[^ ^28^ ^]^ NASBA begins by first converting an ssRNA template into a cDNA/RNA duplex by RTase, which also incorporates a transcription promoter region (e.g. T3 or T7) into the complementary DNA (cDNA) by a promoter‐modified forward primer. Then, RNase H degrades the messenger RNA (mRNA) template in the cDNA/RNA duplex, resulting in the formation of a cDNA duplex by a reverse primer. Finally, the cDNA duplex containing the promoter region is transcribed into mRNA by transcriptase, and this cycle repeats to produce more mRNA copies. Typically, NASBA requires a brief initial heating step at 65 °C to unfold the target RNA template, followed by amplification at ≈41 °C for 5–30 mins.^[^ ^28^ ^]^ Single‐stranded, short mRNA products are well suited for rapid detection by using various molecular sensors, such as molecular beacons, strand displacement, or CRISPR/Cas.^[^ ^29^, ^30^ ^]^ Using NASBA, viral RNA can be detected as low as 3 fM for Zika virus,^[^ ^31^ ^]^ and 200 copies/mL (≈0.3 aM) for SARS‐COV‐2 virus according to a recent report.^[^ ^32^ ^]^ Ju et al. enhanced the sensitivity of NASBA by using a nicking and extension chain reaction.^[^ ^33^ ^]^ To detect DNA targets, an additional denaturation step is required to generate the initial ssDNA template for NASBA amplification.^[^ ^34^ ^]^ Optimizing primers and running protocols will be crucial for improving amplification accuracy, as mRNA production can be more messy than DNA replication and may contain more impurities. “Loop‐Mediated Isothermal Amplification (LAMP)” is a cost‐effective and easy‐to‐use method that can be performed in a single tube to amplify target sequences at a stable temperature of ≈65 °C.^[^ ^35^ ^]^ In Figure 2B, LAMP uses a set of four unique primers (two inner and two outer) to identify six distinct regions on the template DNA.^[^ ^36^ ^]^ A strand‐displacing polymerase (SDP) initiates the synthesis and produces a dumbbell‐shaped amplicon through the formation of internal loops (e.g. F1/F1C and B1/B1C).^[^ ^37^ ^]^ Between the two loops lies a ssDNA amplicon that can be detected using various specially designed probes (e.g. molecular beacon) and sensing circuits that generate colorimetrical, fluorescent, or electrical signals.^[^ ^37^ ^]^ The dumbbell‐shaped amplicon can serve as a new template for SDP to replicate, releasing the loop structure during replication and resulting in exponential amplification. Due to its one‐tube operation, LAMP has been widely reported to detect pathogens, like SARS‐COV‐2, HCV, or Influenza virus.^[^ ^38^, ^39^ ^]^ LAMP requires heating instrumentation that can be miniaturized for field applications.^[^ ^40^, ^41^ ^]^ Primer design tools have been developed to help generate primers for broad use, such as MorphoCatcher.^[^ ^42^ ^]^ “Recombinase Polymerase Amplification (RPA)” has gained significant attention and adoption during the COVID‐19 pandemic, due to its advantages of simple primer design, ease of operation, and rapid amplification at a relatively lower temperature (between 37 °C to 42 °C).^[^ ^43^ ^]^ In Figure 2C, RPA uses ssDNA‐binding proteins (SSB) to destabilize the dsDNA template, and facilitate the insertion of short primers into the template DNA end. The method also employs a SDP polymerase to carry out the synthesis, producing dsDNA amplicons up to 1000 nucleotides long.^[^ ^39^ ^]^ RPA generally requires primers that are 30–35 nucleotides long, which is longer than primers used in PCR. The PrimedRPA, a publicly available Python‐based coding package, can be utilized for automating the design and optimization of RPA primers.^[^ ^44^ ^]^ Users may also use proven PCR primes with extended length for RPA. dsDNA amplicons produced by RPA can be detected in real‐time using nonspecific intercalating dyes (e.g., SybrGreen)^[^ ^45^, ^46^ ^]^ or specially designed probe kits (e.g., TwistAmp nfo kit).^[^ ^39^ ^]^

**Table** **1** summaries the major IA techniques reported over the past few decades, including NASBA,^[^ ^28^ ^]^ LAMP,^[^ ^36^ ^]^ RPA,^[^ ^43^ ^]^ rolling‐circle amplification (RCA),^[^ ^47^ ^]^ helicase‐dependent amplification (HDA),^[^ ^48^ ^]^ strand displacement amplification (SDA),^[^ ^49^ ^]^ cross‐priming amplification (CPA),^[^ ^50^ ^]^ multiple displacement amplification (MDA),^[^ ^51^ ^]^ signal mediated amplification of RNA technology (SMART),^[^ ^52^ ^]^ and polymerase spiral reaction (PSR).^[^ ^53^ ^]^ For IA methods that produce dsDNA amplicon, a common, subsequent step is to convert dsDNA into ss‐mRNA for the compatibility with various signal‐generating mechanisms.^[^ ^54^, ^55^ ^]^ In Figure 2D, the forward primer is modified by adding a promoter extension (e.g. T7), that enables promoter‐initiated mRNA production from a dsDNA template. This mRNA production provides additional amplification beyond dsDNA amplification as one dsDNA template can be transcribed into multiple copies of mRNA. mRNA molecules are also compatible with various sensing platforms, such as SHERLOCK^[^ ^54^ ^]^ and REVEALR.^[^ ^55^ ^]^ However, a drawback of this mRNA production is the increased impurities that can arise during the transcription process, potentially compromising detection accuracy. Alternatively, dsDNA amplicons can be directly converted to ssDNA by using an exonuclease to selectively degrade one strand in the DNA duplex. In Figure 2E, lambda exonuclease (λexo) degrades dsDNA starting from the 5’‐phosphorylated (Phos) end to the 3’ end, while non‐phosphorylated DNA is degraded at a much slower rate.^[^ ^56^ ^]^ To protect a DNA strand from digestion, a phosphorothioate (PS) group can be modified to the 5’end to inhibit the degradation by λexo.^[^ ^57^ ^]^ T7 exonuclease (T7exo) also begins the degradation of dsDNA from the 5’ end and can be inhibited by a 3’ overhang or a 5’ PS modification.^[^ ^37^ ^]^ Additionally, direct amplification of RNA has been reported by using RNA‐dependent RNA polymerase for high throughput screening of SARS‐COV‐2 viral infections.^[^ ^58^ ^]^

**Table 1:** Summary of major IA methods for running temperature, amplicon product and citation results.

IA technique	Temperature	Amplicon product	Google Scholar Citation (2014 – 2024)
NASBA^[^ ^28^ ^]^	first 65 °C, then 41 °C	mRNA	5,470
LAMP^[^ ^36^ ^]^	65 °C	ssDNA with dumbbell shape	25,900
RPA^[^ ^43^ ^]^	37–42 °C	dsDNA	13,500
RCA^[^ ^47^ ^]^	30–65 °C	long ssDNA with repeating regions	18,200
HDA^[^ ^48^ ^]^	65 °C	dsDNA	2,530
SDA^[^ ^49^ ^]^	30–55 °C	dsDNA	6,730
CPA^[^ ^50^ ^]^	30–65 °C	dsDNA	899
MDA^[^ ^51^ ^]^	30–40 °C	dsDNA	5,900
SMART^[^ ^52^ ^]^	37–41 °C	mRNA	155
PSR^[^ ^53^ ^]^	55–60 °C	spiral DNA structure	433

Although current reagents for IA kits could be more expensive than PCR kits, the total cost of an assay should include: 1) reagents, 2) instruments, 3) facilities, and 4) personnel. For PCR, the assay cost should account for all four components, not just the cost of reagents. In contrast, for an IA‐based assay, the costs associated with instruments, facilities, and personnel are significantly reduced. Furthermore, as isothermal amplification becomes more widely adopted, the production scale of reagents is expected to increase, which will also lower the cost per unit assay. Overall, IA techniques could beat conventional PCR for fast speed and simplicity, making them more suitable for POC applications. However, current IA methods do not provide precise quantification of analytes due to the continuous, non‐discrete amplification cycles that occur under isothermal conditions. Therefore, IA‐based assays are primarily used for screening and qualitative testing.

**Table 2:** Overview of isothermal nucleic acid detections discussed in the maintext.

Isothermal nucleic acids detection	Advantages	Disadvantages or Limitation
Molecular beacons^[^ ^19^ ^]^	High sensitivity and specificity via targeted hybridization, real‐time monitoring, multiplexing, quantitative analysis.	Expensive labelled probes, require sequence optimization, lack of additional signal amplification, difficulty in detecting low‐abundance targets.
Toehold‐mediated strand displacement^[^ ^20^ ^,^ ^66^ ^]^	High sensitivity, target‐specific, real‐time monitoring, adaptability for various targets, multiplexing.	Require careful design and optimization, sensitive to conditions, limited dynamic range, expensive labelled probes.
Hybridization chain reaction (HCR)^[^ ^67^ ^]^	Enzyme‐free signal amplification, high sensitivity, target‐specific, real‐time monitoring, multiplexing.	Nonspecific reaction, require careful design and optimization, sensitive to conditions, expensive labelled probes, slow kinetics, qualitative not quantitative.
Catalytic hairpin assembly (CHA)^[^ ^72^ ^]^	Enzyme‐free signal amplification, high sensitivity, target‐specific, modular design for various targets, real‐time monitoring, multiplexing.	Nonspecific signals, require careful design and optimization, sensitive to conditions, qualitative not quantitative, expensive labelled probes.
Competitive “sink”^[^ ^78^ ^]^	Increased specificity against mutations, reduced background noise, detect low‐abundance target, compatible with PCR.	Complex design and optimization, qualitative not quantitative, sink may interact with target.
DNAzyme^[^ ^111^ ^,^ ^115^ ^]^	High sensitivity by catalytic amplification, target‐specific, protein‐free, rapid, real‐time, multiplexing.	Require careful design and optimization, nonspecific signals, sensitive to conditions, expensive labelled probes, qualitative not quantitative.
“C‐Ag‐C" Urease assay^[^ ^88^ ^]^	High sensitivity by enzyme‐based signal amplification, rapid reaction, visible color detection, low cost.	Require careful design and optimization, nonspecific reactions, interference with various metal ions, assay sensitive to pH, qualitative not quantitative.
DNA mediated proximity‐assembly^[^ ^21^ ^]^	High sensitivity by enzyme‐based signal amplification, rapid detection, adaptable to various targets, semi‐quantitative.	May require bioconjugation, enzyme and reagents could be sensitive to conditions.
Riboregulator gen circuits^[^ ^86^ ^,^ ^87^ ^]^	High sensitivity and specificity, modular design adaptable to various targets, improved reagent stability by frozen drying.	Require careful design and optimization, nonspecific reactions, complex in vitro translation system.
Nicking enzyme signal amplification^[^ ^90^ ^,^ ^91^ ^]^	High sensitivity by enzyme‐based signal amplification, rapid detection.	Nicking enzyme optimization, complex enzyme circuits, expensive enzyme and reagents, sensitive to conditions, nonspecific amplification.
CRISPR‐Cas^[^ ^23^ ^,^ ^54^ ^]^	Ultrahigh sensitivity, sequence specific, multiplexing, modular design adaptable to various targets and platforms, rapid.	Costly CRISPR enzyme and RNA, reagent instability and sensitive to conidiations, qualitative not quantitative.

## 3 Nucleic Acid Hybridization Circuits for Signal Amplification and Ultra‐Specific Detection

**Figure 3:**
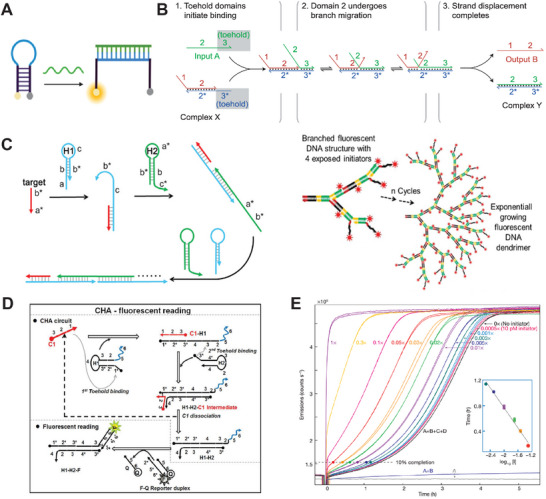
Nucleic acid hybridization circuits for signal amplification. A) Molecular beacon with a pair of fluorophore and quencher. B) Toehold‐mediated strand displacement. Reproduced with permission.^[^ ^20^ ^]^ Copyright 2011, Springer Nature. C) Hybridization chain reaction (left) and branched HCR (right). Reproduced with permission.^[^ ^68^ ^]^ Copyright 2021, American Chemical Society. D) Catalytic hairpin assembly. Reproduced with permission.^[^ ^72^ ^]^ Copyright 2013, American Chemical Society. E) Exponential amplification of fluorescent signal using CHA. Reproduced with permission.^[^ ^73^ ^]^ Copyright 2008, Springer Nature.

Enzyme‐free signal production is gaining attention for POC applications, as it could overcome the drawbacks of expensive enzyme reagents and issues related to storage instability. Molecular beacons (MBs) are one of the early examples of nucleic acid reaction circuits designed for simple signal reporting.^[^ ^19^ ^]^ In **Figure** **3**A, a typical MB uses a hairpin structure to bring together a pair of fluorophore and quencher (e.g. Cy3 and Black Hole Quencher‐2) that are attached on 5’ or 3’ ends of a DNA duplex stem, resulting in quenched fluorescence. When a target strand hybridizes to the hairpin, it disrupts the duplex stem, separating the fluorophore from the quencher, resulting in more fluorescent signals. Some studies observed that certain fluorophores (e.g. fluorescein) can also be quenched by DNA duplexes,^[^ ^59^, ^60^ ^]^ ssDNA overhangs,^[^ ^60^, ^61^ ^]^ or unique secondary structures like G‐quadruplex,^[^ ^62^ ^]^ which allows for the design of simpler versions of MBs without a quencher. MBs are well suited for detecting ssDNA or ssRNA targets, and have been widely adopted in combination with IA methods.^[^ ^30^, ^63^ ^]^ MBs could also be used to detect dsDNA targets with the help of transcription activator‐like effectors.^[^ ^64^ ^]^ Additionally, multiplex detection using MBs has been reported.^[^ ^65^ ^]^

Toehold‐mediated strand displacement (TMSD) plays a crucial role in nucleic acid hybridization circuits for sensing a target strand. In Figure 3B, TMSD is initiated by an unpaired toehold binding to a ssDNA or ssRNA target. This is followed by a branched migration, during which the target strand displaces a reporter strand from an existing hybridized complex.^[^ ^20^ ^]^ This process is driven by the thermodynamic competition, where the newly formed duplex is more thermally stable than the original duplex, resulting in an overall negative ΔG. TMSD has been used to engineer complex nucleic acid hybridization, assembly, and computation circuits to enhance sensitivity and specificity of detection.^[^ ^66^ ^]^ In Figure 3C, the Pierce lab invented the hybridization chain reaction (HCR), which used an initiator strand to trigger the hybridization of multiple hairpins, forming a linear and long duplex structure.^[^ ^67^, ^68^ ^]^ By labeling hairpins with fluorophores, HCR generated fluorescent amplification from multiple reporter dyes when a target strand acts as an initiator.^[^ ^69^ ^]^ Later, branched HCR was reported for producing even greater signal amplification than the linear HCR and enabling multiplexed detection.^[^ ^70^, ^71^ ^]^ In Figure 3D, catalytic hairpin assembly (CHA) is another signal amplification circuit by producing multiple copies of short duplexes, instead of a long linear duplex.^[^ ^72^ ^]^ CHA uses a single strand to catalyze the hybridization of two hairpins (H1 and H2), forming a short H1/H2 duplex. The catalytic strand is restored when the H1/H2 duplex is formed, allowing it to trigger another assembly cycle. Figure 3E demonstrates the exponential amplification of the fluorescent signal through the use of an initiator strand to trigger catalytic hairpin assembly.^[^ ^73^ ^]^ Over the past decade, various DNA hybridization circuits have been reported to enhance fluorescence for nucleic acid detection and imaging in diseases.^[^ ^74^, ^75^, ^76^ ^]^ Though promising, HCR and CHA require optimization of sequences and conditions to minimize background signals from nonspecific assembly. Additionally, the catalytic assembly process can take several hours to enrich the signal, which is much slower than enzyme‐based signal amplification, thus limiting its application for rapid and precise detection.

**Figure 4:**
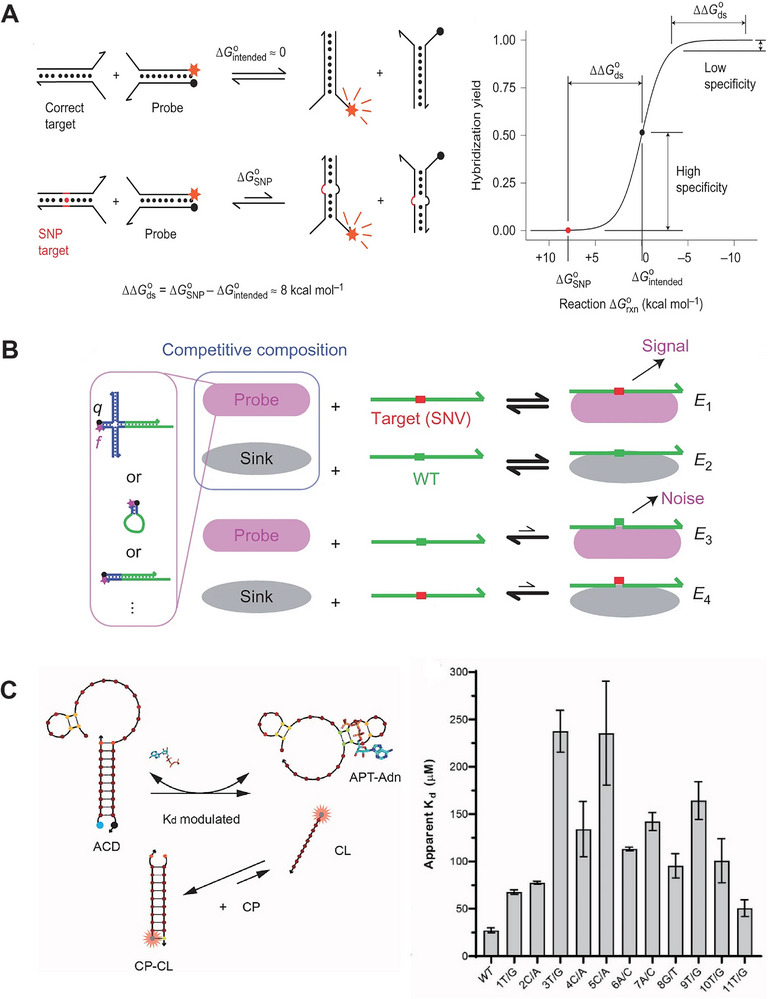
Nucleic acid hybridization circuits for ultra‐specific detection of single‐ nucleotide mutation. A) Toehold exchange‐based reaction to detect single‐nucleotide mismatch. Reproduced with permission.^[^ ^77^ ^]^ Copyright 2013, Springer Nature. B) A competitive “sink” to increase the specificity of strand displacement to detect a SNV. Reproduced with permission.^[^ ^78^ ^]^ Copyright 2015, Springer Nature. C) Competitive aptamer switch to modulate binging affinity and sense single‐nucleotide mutation. Reproduced with permission.^[^ ^81^ ^]^ Copyright 2023, KeAi Publishing.

Nucleic acid hybridization circuits are also used to enhance specificity for detecting mutations within sequences, especially single‐nucleotide variants (SNVs). SNVs refer to the alteration of a single nucleotide at specific genomic locations, which are associated with various human diseases, like cancers. Early detection of rare SNVs can significantly benefit patient treatment and economic outcomes. By designing specific nucleic acid circuits, it becomes possible to distinguish small ΔG variations in hybridization caused by SNVs and generate signals for detecting them. In **Figure** **4**A, Chen et al. described a method using toehold exchange‐based reaction to identify SNVs, where target‐probe hybridization triggered a TMSD that disrupts the dye‐quencher interaction, leading to increased fluorescence.^[^ ^77^ ^]^ This study revealed that the specificity for SNV detection was affected by the overall ΔG of the intended strand exchange, with the optimal specificity reached when the exchange occurred at nearly zero ΔG. Based on this finding, the Zhang group further developed a competitive “sink” to improve TMSD specificity for SNV detection. In Figure 4B, a “sink” strand competes with the probe structure for binding to a wildtype (WT) target, effectively blocking WT‐probe interactions.^[^ ^78^ ^]^ The SNV target hybridizes to the “sink” less effectively than WT‐sink binding, making it more efficient for binding to the probe structure. This competitive mechanism has been shown to achieve ultra‐specificity in SNV‐probe hybridization, resulting in a sensitivity increase of 200 to 3,000 times compared to WT‐probe hybridization. A rare SNV target as low as 0.01% was detected by using the competitive “sink.”^[^ ^78^ ^]^ Following this, Zhang group developed “Blocker displacement amplification (BDA)” to enrich rare variant sequences in multiplexing detection and PCR reactions.^[^ ^79^, ^80^ ^]^ In Figure 4C, competitive hybridization is used to modulate an aptamer's binding to an adenosine, where variations in ΔG affected the dissociation constant (K_d_).^[^ ^81^ ^]^ The overall ΔG and equilibrium of this competitive aptamer switch were sensitive to single‐nucleotide mutations in a competing strand. Additionally, the aptamer switch for ligand binding could be regulated by toehold exchange reactions.^[^ ^82^ ^]^

## 4 Enzyme‐Based Signal Amplification for Single‐Stranded Nucleic Acids

**Figure 5:**
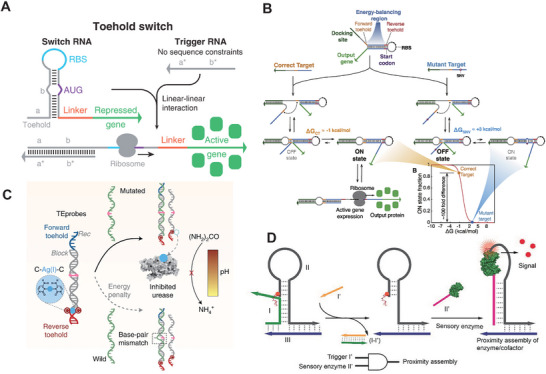
A) A toehold switch‐mediated riboregulatory system for detect a trigger RNA to activate protein expression. Reproduced with permission.^[^ ^22^ ^]^ Copyright 2014, Cell Press. B) Single‐nucleotide specific programmable riboregulators. Reproduced with permission.^[^ ^87^ ^]^ Copyright 2020, Cell Press C) Strand displacement with “C‐Ag‐C” clamp for colorimetric detection of target strands. Reproduced with permission.^[^ ^88^ ^]^ Copyright 2022, Springer Nature. D) DNA hairpin‐mediated proximity‐based assembly circuit. Reproduced with permission.^[^ ^21^ ^]^ Copyright 2018, John Wiley & Sons.

Enzymes are biological catalysts that accelerate chemical reactions with a rate enhancement by millions to billions of times.^[^ ^83^, ^84^ ^]^ Enzyme‐catalyzed reactions are commonly employed in diagnostic assays to amplify detectable signals, thereby increasing the sensitivity. The Collins lab developed riboregulators that incorporate unique RNA sequences to regulate the structure of ribosome binding site in a target gene, allowing for control over post‐transcriptional gene regulation and production of green fluorescent protein (GFP) in cells.^[^ ^85^ ^]^ Later, Green et al. reported de novo‐designed riboregulators that utilized a toehold switch to activate protein translation by recognizing an RNA target. In **Figure** **5**A, the riboregulatory system consists of a toehold, a hairpin‐blocked ribosome‐binding site (RBS), and a repressed gene segment.^[^ ^22^ ^]^ Initially, the hairpin‐blocked RBS cannot bind to a ribosome. However, in the presence of an RNA target, the RNA‐toehold hybridization triggers the opening of the hairpin structure, exposing the RBS for ribosome binding. This activated riboregulatory system produces GFP for fluorescent detection. For convenient use, the toehold‐switch riboregulatory system was freeze‐dried onto a paper disc, which could be activated by rehydrating with an analyte solution to generate fluorescent or colorimetric signals for detection.^[^ ^86^ ^]^ Hong et al. reported an ultra‐specific mechanism of “single‐nucleotide‐specific programmable riboregulators” (SNIPRs) to detect mismatches caused by single‐nucleotide mutations in DNA and RNA sequences.^[^ ^87^ ^]^ In Figure 5B, SNIPRs uses an energy‐balancing region with forward and reverse toeholds to differentiate ΔG variations between a target strand and a mutant strand for binding to the hairpin structure, thereby mediating the “ON” and “OFF” states of protein expression. SNIPRs can identify mismatches in hybridization from a mutant strand because the small, unfavored ΔG of just a few kcal/mol keeps the system in the “OFF” state. The combination of the IA‐based RT‐RPA and SNIPRs demonstrated a portable color detection of various cancer‐associated mutations, and a high sensitivity in detecting viral RNA at concentrations as low as 250 attomolar. Zhang et al. reported a paper‐based method for color detection of viral RNA by utilizing specific “C‐Ag‐C” interaction to mediate urease activity.^[^ ^88^ ^]^ In Figure 5C, strand displacement between a DNA probe (TEprobe) and an RNA target disrupts the “C‐Ag‐C” clamp, releasing Ag^+^ ions to inhibit urease activity, which can be simply detected by pH‐mediated color changes. In Figure 5D, a DNA hairpin‐mediated proximity‐based assembly circuit (DPAC) is used to activate enzyme‐catalyzed biochemical reactions.^[^ ^21^ ^]^ In DPAC, a cofactor is sterically locked by a DNA hairpin, preventing interaction with an enzyme. A target strand triggers a conformational change in the DNA hairpin, exposing the cofactor to allow the co‐assembly of the enzyme and cofactor on a dsDNA scaffold. This proximity assembly enhances the catalytic reaction rate to generate more colorimetric or fluorescent signals. DPAC has also been demonstrated to detect small molecule targets such as adenosine using an aptamer switch.

**Figure 6:**
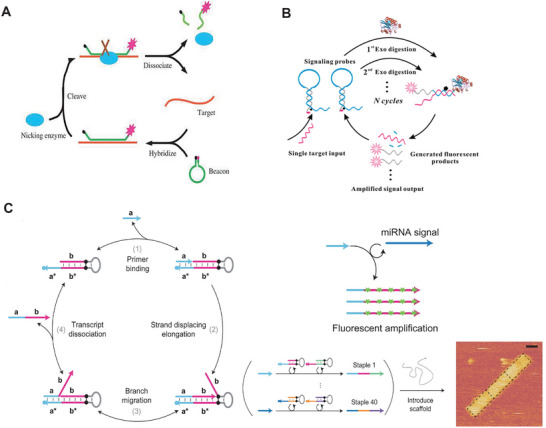
Nicking enzyme signal amplification and primer exchange circuits. A) Working principle for the basic NESA. Reproduced with permission.^[^ ^91^ ^]^ Copyright 2008, Oxford University Press. B) Exonuclease III aided target recycling. Reproduced with permission.^[^ ^94^ ^]^ Copyright 2010, American Chemical Society. C) A primer exchange cascade to catalyze the sequential elongation of a single‐strander primer with prescribed sequences by using hairpin templates. Reproduced with permission.^[^ ^97^ ^]^ Copyright 2023, Springer Nature.

Nicking enzyme signal amplification (NESA) uses a nicking enzyme to exponentially generate reporter strands by recycling a target strand during the reaction.^[^ ^89^, ^90^ ^]^ In **Figure** **6**A, a ssDNA target is first hybridized to a hairpin MB, exposing a nicking site. The nicking enzyme then cleaves the MB strand, releasing fluorescence for detection.^[^ ^91^ ^]^ The ssDNA target is released for binding to the next hairpin MB. To enhance sensitivity, NESA was used to produce G‐quadruplex/hemin (GQH) DNAzyme,^[^ ^92^ ^]^ or to trigger the assembly of gold nanoparticles (GNPs) for colorimetric detection.^[^ ^93^ ^]^ In Figure 6B, exonuclease III (Exo III) assists the signal amplification to detect ssDNA by cleaving the MB strand and releasing fluorescent probes, while simultaneously recycling the ssDNA target.^[^ ^94^ ^]^ Exo III‐assisted reaction has also been used to produce GQH^[^ ^95^ ^]^ and to initiate HCR assembly^[^ ^96^ ^]^ for more versatile detections. In Figure 6C, the Yin lab introduced the concept of primer exchange reaction (PER) cascades that catalyzed the sequential elongation of a single‐stranded primer with prescribed sequences coded by a series of hairpin templates.^[^ ^97^ ^]^ The appended prescribed sequence can facilitate the assembly of DNA origami structures, form a DNAzyme, bind to intercalating dyes, or produce DNA‐metal nanoclusters for detection.^[^ ^97^, ^98^ ^]^ Phosphorothioated (PS) hairpin was also used to assist the polymerase amplification of long, SYBR‐intercalated dsDNA reporters for detection.^[^ ^99^ ^]^

**Figure 7:**
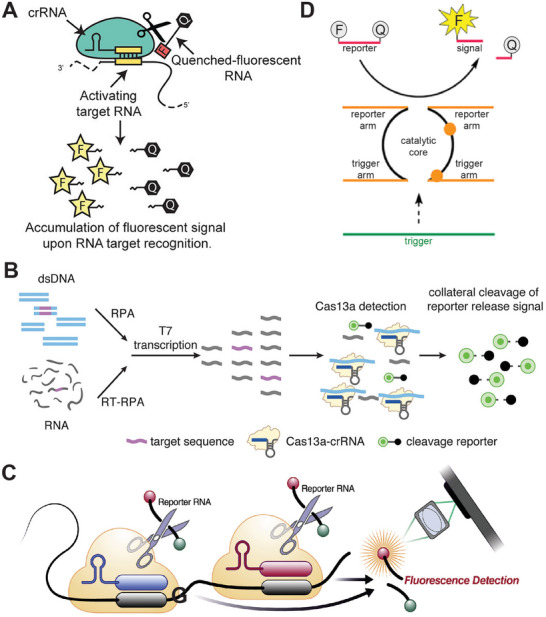
Nucleic acid detection by using CRISPR‐based system and DNAzyme. A) CRISPR‐Cas detection of a target RNA by the activated RNase activity to cleave a fluorescent substrate. Reproduced with permission.^[^ ^104^ ^]^ Copyright 2016, Springer Nature. B) The “SHERLOCK” platform integrates isothermal amplification (e.g. RPA) with CRISPR‐Cas detection. Reproduced with permission.^[^ ^54^ ^]^ Copyright 2019, Springer Nature. C) The use of multiple crRNA sites to improve the sensitivity and specificity for detecting viral RNA. Reproduced with permission.^[^ ^105^ ^]^ Copyright 2021, Cell Press. D) RNA target to trigger the activation of a DNAzyme for reporting fluorescence signals. Reproduced with permission.^[^ ^115^ ^]^ Copyright 2022, American Chemical Society.

Over the past decade, the discovery of “Clustered Regularly Interspaced Short Palindromic Repeats” (CRISPR) and RNA‐guided CRISPR‐associated proteins (Cas) has brought new insights into gene therapy^[^ ^100^ ^]^ and nucleic acid detection.^[^ ^101^, ^102^ ^]^ The Zhang and Koonin labs reported a C2c2 Cas protein (later called Cas13a) which performed RNase activity triggered by the binding of a single effector RNA.^[^ ^103^ ^]^ In the same year, the Doudna lab utilized the RNase activity of CRISPR‐C2c2 to detect RNA targets.^[^ ^104^ ^]^ In **Figure** **7**A, the Cas13a‐CRISPR RNA (crRNA) complex is activated upon binding to a target RNA, showing RNase activity. The activated Cas13a‐crRNA complex cleaves an oligonucleotide (oligo) substrate, releasing fluorescence for detection. To enhance detection sensitivity, the “SHERLOCK” platform was developed by combining CRISPR‐Cas with IA methods (Figure 7B), allowing for nucleic acid detection within one hour at attomolar sensitivity.^[^ ^54^ ^]^ The results from this assay can be easily read by a later‐flow strip, making it well‐suited for POC diagnosis.^[^ ^54^, ^152^ ^]^ In Figure 7C, Fozouni et al. employed multiple crRNA binding sites in conjugation with Cas proteins to detect several target regions on SARS‐COV2 viral RNA. It boosted the sensitivity to ≈100 RNA copies/µL without requiring pre‐amplification steps.^[^ ^105^ ^]^ Jiao et al. discovered the formation of noncanonical crRNA (ncrRNA) when cellular RNA hybridized with trans‐activating crRNA (tracrRNA) and Cas9 enzymes.^[^ ^106^ ^]^ These ncrRNAs could direct Cas9 to complementary DNA targets for cleavage. Utilizing this mechanism, the LEOPARD platform was demonstrated for the simultaneous detection of multiple viral RNAs by gel analysis, distinguishing different variants of SARS‐COV2. Several recent reviews have summarized the advancements in CRISPR‐based diagnostics and their applications.^[^ ^23^, ^107^, ^108^ ^]^ In some applications, CRISPR technology was integrated into portable devices for POC tests,^[^ ^109^ ^]^ such as a portable glucose meter‐based platform for daily screening of COVID‐19.^[^ ^110^ ^]^

Deoxyribozyme (DNAzyme), is a unique type of oligos that forms pocket‐like structures capable of catalyzing chemical reactions.^[^ ^111^ ^]^ DNAzymes are generally more stable than ribozymes, and show promise in molecular diagnostics,^[^ ^112^ ^]^ therapeutics,^[^ ^113^ ^]^ and environmental sensing.^[^ ^114^ ^]^ In **Figure** 7D, a platform called “RNA‐Encoded Viral Nucleic Acid Analyte Reporter” (REVEALR) is used to detect viral RNAs from different variants for SARS‐COV2 genotyping.^[^ ^115^ ^]^ This platform was based on a multicomponent X(Xeno)NAzyme optimized from the DNAzyme of X10–23, which cleaved a substrate oligo by binding to a target viral RNA.^[^ ^55^ ^]^ By combining this with IA methods, such as RPA, REVEALR reached a detection limit of <20 aM (∼10 copies/µL) in less than one hour. An advanced version of REVELR was able to directly detect viruses collected from nasopharyngeal swabs through digital imaging of signals in droplets and AI‐assisted readout.^[^ ^116^ ^]^ Recent reviews have detailed advancements in DNAzyme‐based diagnostic tools.^[^ ^117^, ^118^, ^119^ ^]^ **Table** 2 summarizes various isothermal assays for detecting nucleic acids with reporting colorimetric or fluorescent signals.

## 5 Detection Platforms for POC Diagnosis of Nucleic Acids

The development and implementation of effective detection platforms are essential for advancing POC medical diagnostics and public health, particularly in alignment with ASSURED criteria.^[^ ^7^ ^]^ The ideal platform should be affordable, accessible, user‐friendly, sensitive, and specific, enabling a rapid response to public health emergencies and challenges. Below, we briefly discuss major detection platforms for rapid nucleic acid detection, including lateral flow assay, paper disc, microfluidics, electrochemical sensors, and portable and wearable devices.

**Figure 8:**
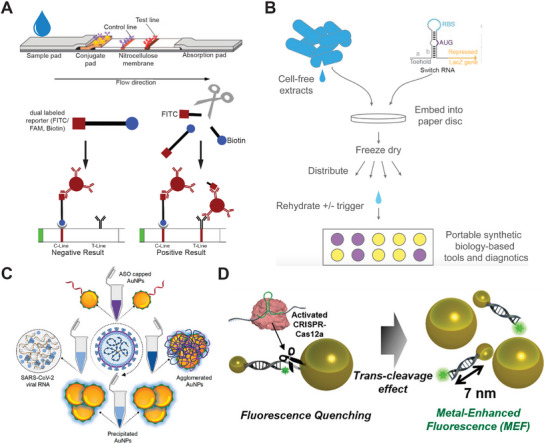
Technology platforms that offer simple operation and rapid signal reporting for POC applications. A) Principle of a lateral flow assay on a strip consisting of multiple zones. Reproduced with permission under a Creative Commons CC‐BY license.^[^ ^120^ ^]^ Copyright 2022, Frontiers. Copyright 2024, Milenia Biotec. B) A Paper disc‐based riboregulatory gene circuit for detecting RNA. Reproduced with permission.^[^ ^86^ ^]^ Copyright 2014, Cell Press. C) Target strands trigger the agglomeration of GNPs for visible color detection. Reproduced with permission.^[^ ^131^ ^]^ Copyright 2020, American Chemical Society. D) GNP‐assisted metal‐enhanced fluorescence for a CRISPR‐Cas12a based nucleic acid biosensor. Reproduced with permission.^[^ ^134^ ^]^ Copyright 2021, American Chemical Society.

A lateral flow assay (LFA), also referred to as a lateral flow immunochromatographic assay, utilizes capillary force to transport liquid samples and reagents across multiple zones on a pad, capturing both the sample and signal probes based on principles similar to affinity chromatography.^[^ ^120^, ^121^ ^]^ Due to its ease of use, rapid results (under half an hour), and low cost, LFAs have been widely adopted for POC testing in both laboratory and home settings. In **Figure** **8**A, a typical LFA is conducted on a strip that includes several zones: 1) a sample pad for depositing liquid sample, 2) a conjugated pad containing color reagents (e.g., antibodies‐modified nanoparticles) that interact with the target for detection, 3) a nitrocellulose membrane featuring at least one test line and one control line, and 4) an absorbent pad that draws the liquid along the strip.^[^ ^120^ ^]^ For instance, a biotin/FITC dual‐labeled oligo probe first binds to an anti‐FITC gold nanoparticle (GNP) at the conjugate pad, which is then captured by streptavidin fixed on the control line, resulting in a visible color change at the control line. Very few biotin/FITC‐oligo/GNPs remain to migrate toward the test line without causing an obvious color change at the line, indicating a negative result. However, if there is a target‐triggered cleavage of oligo probes by an assay (e.g. Cas9 protein or DNAzyme),^[^ ^54^, ^55^, ^152^ ^]^ FITC‐half oligo/GNPs will pass the control line and migrate to the test line, where they are captured by anti‐GNP antibodies fixed on the test line. This results in a color change on the test line, indicating a positive result. LFAs can also use fluorescence signals that are detectable by portable and mobile devices.^[^ ^105^ ^]^ In addition to using affinity reagents, some studies have employed specific DNA‐DNA hybridizations to capture signals on the LFA strip.^[^ ^122^, ^123^ ^]^ To ensure accuracy for LFAs, it is crucial to optimize the concentrations of probe strands and signal reagents (e.g. GNPs) to minimize nonspecific false positives, which could arise from insufficient reporter strands or high dose‐induced Hook effect.^[^ ^124^ ^]^ For highly sensitive detection, LFAs are also combined with isothermal technologies, such as LAMP,^[^ ^125^ ^]^ RPA,^[^ ^126^ ^]^ and RCA.^[^ ^127^ ^]^ Paper disc‐based detection represents another inexpensive and simple platform, enabling versatile, cost‐effective, and user‐friendly applications. In Figure 8B, Pardee et al. reported a paper disc‐based, toehold‐mediated riboregulator circuit for detecting nucleic acid targets.^[^ ^86^ ^]^ To prepare this, a few droplets of a cell‐free gene circuit were deposited on a cellulose filter paper, and then rapidly freeze‐dried. The freeze‐dried discs were stable for storage and transport. The activity of this dried, cell‐free gene circuit can be simply restored by adding analyte solution, allowing the targets‐triggered gene translation to produce fluorescent signal^[^ ^86^ ^]^ or visible color change.^[^ ^31^ ^]^ These paper‐based gene networks have been demonstrated for the quick detection of viral RNA from Ebola virus,^[^ ^86^ ^]^ Zika virus,^[^ ^31^ ^]^ HIV,^[^ ^87^ ^]^ and SARS‐CoV‐2.^[^ ^128^ ^]^

Plasmonic nanoparticles are often used as indicators to generate visible signals for “naked‐eye” detection in POC testing.^[^ ^129^, ^130^ ^]^ In Figure 8C, specific hybridizations between viral RNA and anti‐sense oligos trigger the agglomeration of smaller GNPs into large particle complexes, resulting in a red shift of absorbance for GNPs solution and a visible precipitate indicating the presence of viral RNA.^[^ ^131^ ^]^ Other “naked‐eye” assays have also been reported to detect specific RNA hybridizations through the induced aggregation of GNPs, which causes a visible color change from red/pink to blue,^[^ ^132^ ^]^ or through the formation of nano “sticky balls” between magnetic particles and GNPs.^[^ ^133^ ^]^ GNPs and quantum dots (QDs) are also used for producing fluorescent signals for more sensitive detection. In Figure 8D, GNPs‐assisted, metal‐enhanced fluorescence (MEF) is employed to enhance the sensitivity of a CRISPR‐Cas12a based nucleic acid biosensor.^[^ ^134^ ^]^ This GNPs‐assisted MEF includes a pair of GNPs with sizes of 20 and 60 nm, linked together by a long dsDNA and a short ssDNA carrying a quenched FITC positioned at ≈2 nm away from the 60‐nm GNP. The target‐activated CRISPR‐Cas12a complex cleaves ssDNA and releases FITC from 60‐nm GNPs, resulting in strong MEF signals.

**Figure 9:**
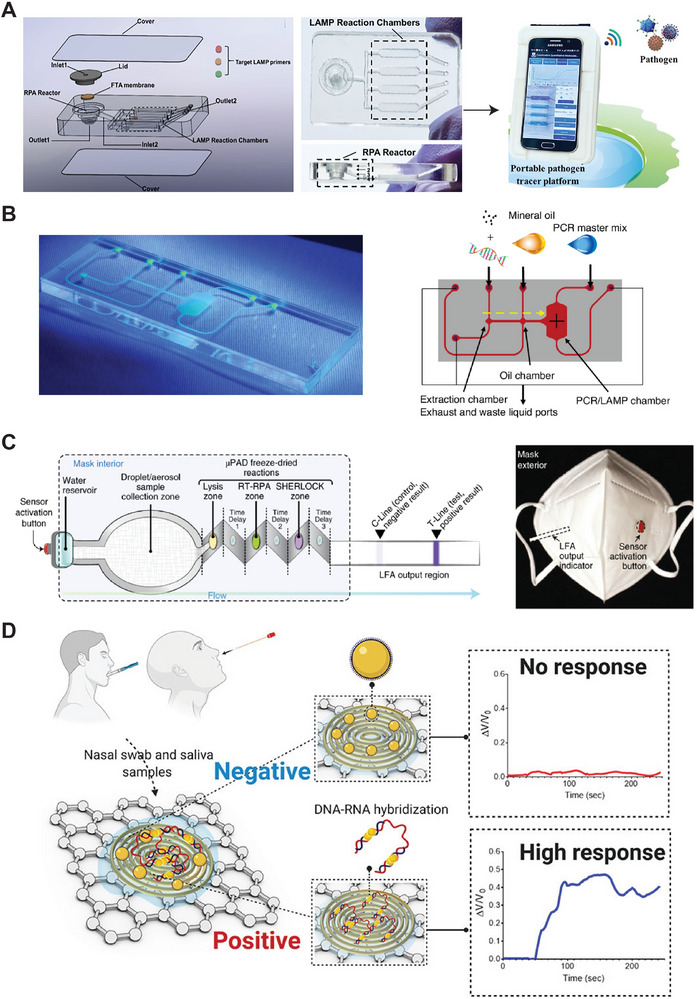
Portable and wearable diagnostic solutions using lab‐on‐a‐chip (LOC) system. A) 3D‐printed, microfluidic device integrates nucleic acid extraction and isothermal amplification on a multi‐layer platform. Reproduced with permission.^[^ ^138^ ^]^ Copyright 2021, Elsevier. B) A microfluidic assay platform featuring integrated pathways for extraction and miniaturized RT‐LAMP or RT‐qPCR for RNA detection. Reproduced with permission under a Creative Commons Attribution 4.0 International License.^[^ ^153^ ^]^ Copyright 2024, Springer Nature. C) A prototype LOC facemask for real‐time virus detection. Reproduced with permission.^[^ ^154^ ^]^ Copyright 2021, Springer Nature. D) A paper‐based electrochemical sensor readable by a handheld device. Reproduced with permission.^[^ ^142^ ^]^ Copyright 2020, American Chemical Society.

Microfluidic devices handle small volumes of analytes and reagents in micrometer‐scale channels, allowing for integration and miniaturization on a microfabricated, lab‐on‐a‐chip (LOC) system.^[^ ^135^, ^136^, ^137^ ^]^ In **Figure** **9**A, a 3D‐printed, microfluidic device integrates nucleic acid extraction with an amplification assay of RPA and LAMP through multi‐layer fabrication, making it suitable for use.^[^ ^138^ ^]^ The device enabled sensitive and multiplexed colorimetric detection of pathogen nucleic acids (e.g. SARS‐COV‐2, *E. coli*) from wastewater for environmental health monitoring. Figure 9B describes a microfluidic‐based assay platform that integrates the extraction and purification of nucleic acids, alongside miniaturized amplifications like RT‐LAMP or RT‐qPCR.^[^ ^153^ ^]^ This microfluidic chip performed core operations within a portable device for POC testing, achieving a low LoD of less than 300 RNA copies of SARS‐COV‐2, and a low‐cost assay ≈$ 9.5 each. Paper microfluidics can offer even low‐cost fabrication for assays. Julien et al. utilized paper microfluidics for sample preparation and mixing, as well as for controlling sample flow through multiple lateral flow channels to enable multiplex LAMP‐based nucleic acid detection for malaria.^[^ ^139^ ^]^ In Figure 9C, a prototype, LOC‐like facemask was demonstrated for the real‐time virus detection by integrating sample collection, cell lysis, isothermal nucleic acid assays (e.g. SHERLOCK), and a LFA strip for visible signal readout.^[^ ^154^ ^]^ Such a wearable POC device is particularly useful for monitoring viral infections in high‐risk and contamination areas, such as emergency rooms, hospital clinics, and rapid testing sites.

**Figure 10:**
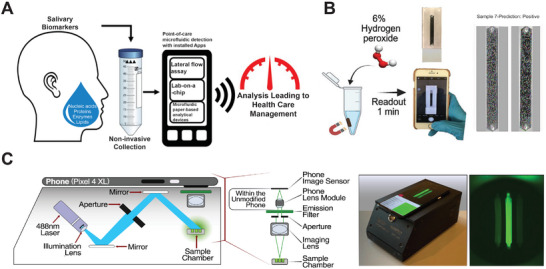
Mobile‐linked POC diagnostics for nucleic acids. A) A conceptual smartphone‐connected POC test. Reproduced with permission under the Creative Commons Attribution License.^[^ ^144^ ^]^ Copyright 2023, Ivyspring International Publisher. B) A smartphone to capture and analyze assay results. Reproduced with permission.^[^ ^157^ ^]^ Copyright 2021, John Wiley & Sons. C) A mobile phone microscopy setup for detecting CRISPR‐Cas13a assay results. Reproduced with permission.^[^ ^105^ ^]^ Copyright 2021, Cell Press.

Electrochemical biosensors are gaining attention in diagnostics due to their unique ability to convert biological interactions into electrical signals, which can be integrated with various electronic devices, including wearable technology.^[^ ^140^ ^]^ Electrochemical sensors have shown high efficiency, low cost, and ease of operation in detecting nucleic acids.^[^ ^141^ ^]^ In Figure 9D, Alafeef et al. reported a paper‐based electrochemical sensor for detecting viral RNA from SARS‐COV2 virus.^[^ ^142^ ^]^ The sensor used anti‐sense ssDNA capped GNPs to capture viral RNA onto a graphene surface, resulting in an increased output voltage. The signal response was read out by a handheld device within just 5 minutes of incubation, achieving a LoD of ≈6.9 copies/µL (≈11 aM) for viral RNA. Additionally, electrochemical sensors were integrated into a lab‐on‐a‐chip device that automatedly extracted, concentrated, and amplified viral RNA from unprocessed saliva.^[^ ^155^ ^]^ Mobile technology has significantly impacted daily life through devices like smart phones, tablets, and smartwatches. Mobile‐linked POC devices hold great potential for enhancing community health and facilitating efficient communication between patients and healthcare professionals.^[^ ^143^, ^156^ ^]^ Smartphones equipped with high‐quality optical cameras and sensors could serve as alternatives to advanced laboratory spectrometers for reading and analyzing results.^[^ ^156^ ^]^ In **Figure** **10**A, miniaturized sensing platforms, such as LFA and LOC systems, can be connected to a smartphone, enabling users to quickly read and analyze assay results for POC testing.^[^ ^144^ ^]^ In Figure 10B, a simple example involves using a smartphone to capture images of oxygen‐generating bubbles in a CRISPR‐Cas based enzyme assay, followed by analyzing the results for SARS‐COV‐2 infection using mobile apps.^[^ ^157^ ^]^ In Figure 10C, Fozouni et al. described a miniaturized microscope module that integrated a laser diode, optical lens, and filters. The module can be attached to a smartphone to capture fluorescence signals from a CRISPR‐Cas13a assay for viral RNA detection.^[^ ^105^ ^]^ Recent advancements in smartphone‐based nucleic acid diagnostics have been discussed in various publications.^[^ ^145^, ^158^ ^]^

Among the POC platforms discussed above, LFA and paper disc gene circuits provide simple and rapid detection of nucleic acids. They are user‐friendly and require minimal accessories when paired with colorimetric methods. However, careful optimization is necessary to reduce the occurrence of false positives and negatives. Additionally, LFA and paper disc methods lack the integrated systems needed for extraction, amplification, and detection on a single strip. Microfluidic systems or LOC offer the advantage of integrating complex processes from sample collection to final signal reporting. However, the manufacturing process for microfluidic chips is complex and more costly than other POC tests. Recent advancements in paper microfluidics have the potential to simplify production and reduce costs,^[^ ^139^ ^]^ but the effectiveness of these paper‐based POC tests has yet to be demonstrated. Electrochemical biosensors are advantageous due to their high sensitivity, miniaturization, and wearability. Nonetheless, electrical signals can be disrupted by environmental electromagnetic fields, which may compromise signal specificity. Mobile and wearable technologies hold promise for the future of point‐of‐care diagnostics, but further efforts are needed to adapt various isothermal methods for use with these devices.

## 6 Conclusion

In summary, significant progress has been made in isothermal technologies, sensing circuits, and assay platforms for the detection of single‐stranded nucleic acids over the past few decades. These technology breakthroughs have promoted the development of simple and reliable nucleic acid tests for POC applications, which will lead to transformative advancement in medical diagnosis. Though promising, the journey toward true POC nucleic acid tests at the place of patient care is still underway. Isothermal nucleic acid assays are primarily used in clinics and laboratories by trained personnel, even though the procedures and operations have been simplified. Limitations remain that hinder broader applications for at‐home POC and OTC use for community health.

The main obstacle is the difficulty and complexity of integrating the multiple steps required for nucleic acid detection, which includes extraction/purification, amplification, and detection. Many rapid nucleic acid assays published to date are designed for use with extracted and purified samples, requiring centrifugation or magnetic separation. A more simplified extraction method, such as one‐step, syringe filtration,^[^ ^146^, ^147^ ^]^ could be more beneficial for POC use. Some portable diagnostic devices, like Abbott's ID NOW^TM^ platform, can work directly with swab samples to rapidly detect disease nucleic acids by incorporating all necessary steps into a benchtop device. Such benchtop POC solutions are welcomed for use in clinic offices or can be made portable for fieldwork when equipped with rechargeable batteries. To enhance the convenience for at‐home POC and OTC use, future efforts should be focused on the low‐cost, miniaturization of a “one‐step” operation, integrating steps from sample collection to result readout. Additionally, developing battery‐powered devices that are compact and rugged would be beneficial for POC applications. It is also important to note that isothermal assays are best suited for qualitative detection for screening and do not provide precise quantification of nucleic acid loads. The development of statistical models for performance evaluation could improve both qualitative and semi‐quantitative analyses in isothermal assays. The simultaneous and multiplex detection of various nucleic acids targets is also of great interest to POC diagnosis.^[^ ^106^, ^109^ ^]^

On the other hand, PCR retains its advantages for quantitative analysis, offering high sensitivity and specificity. It has not completely fallen behind in the competition for POC diagnosis. New advancements in optics, electronics, and photothermal materials enable the development of miniaturized and portable PCR. For example, a recent, promising development is plasmonic PCR, which utilizes the high optical absorbance of metallic nanoparticles (e.g. GNPs) or thin plasmonic films to trigger rapid thermocycling within a small volume controlled by light illumination.^[^ ^148^ ^]^

Overall, POC nucleic acid tests have great potential to revolutionize personal diagnostics, patient management, and disease surveillance, ultimately enhancing patient outcomes and community health.

## Conflict of Interest

The authors declare no competing financial interest.

## Author Contributions

All authors contributed to the preparation of the manuscript.
